# Incorporating B cell activating factor (BAFF) into the membrane of rabies virus (RABV) particles improves the speed and magnitude of vaccine-induced antibody responses

**DOI:** 10.1371/journal.pntd.0007800

**Published:** 2019-11-14

**Authors:** Joseph R. Plummer, James P. McGettigan

**Affiliations:** 1 Department of Microbiology and Immunology, Thomas Jefferson University, Philadelphia, Pennsylvania, United States of America; 2 Jefferson Vaccine Center, Thomas Jefferson University, Philadelphia, Pennsylvania, United States of America; Universidad Nacional Mayor de San Marcos, PERU

## Abstract

B cell activating factor (BAFF) is a member of the tumor necrosis factor (TNF) superfamily of cytokines that links innate with adaptive immunity. BAFF signals through receptors on B cells, making it an attractive molecule to potentiate vaccine-induced B cell responses. We hypothesized that a rabies virus (RABV)-based vaccine displaying both antigen and BAFF on the surface of the same virus particle would target antigen-specific B cells for activation and improve RABV-specific antibody responses. To test this hypothesis, we constructed a recombinant RABV-based vector expressing virus membrane-anchored murine BAFF (RABV-ED51-mBAFF). BAFF was incorporated into the RABV particle and determined to be biologically functional, as demonstrated by increased B cell survival of primary murine B cells treated *ex-*vivo with RABV-ED51-mBAFF. B cell survival was inhibited by pre-treating RABV-ED51-mBAFF with an antibody that blocks BAFF functions. RABV-ED51-mBAFF also activated primary murine B cells *ex-vivo* more effectively than RABV as shown by significant upregulation of CD69, CD40, and MHCII on the surface of infected B cells. *In-vivo*, RABV-ED51-mBAFF induced significantly faster and higher virus neutralizing antibody (VNA) titers than RABV while not adversely affecting the longevity of the vaccine-induced antibody response. Since BAFF was incorporated into the virus particle and genome replication was not required for BAFF expression *in-vivo*, we hypothesized that RABV-ED51-mBAFF would be effective as an inactivated vaccine. Mice immunized with 250 ng/mouse of β-propriolactone-inactivated RABV-ED51-mBAFF showed faster and higher anti-RABV VNA titers compared to mice immunized with inactivated RABV. Together, this model stands as a potential foundation for exploring other virus membrane-anchored molecular adjuvants to make safer, more effective inactivated RABV-based vaccines.

## Author summary

Over two-thirds of the world’s population lives in regions where rabies is endemic. Each year, more than 15 million people receive multi-dose PEP and over 59,000 people die: one rabies death every 9 minutes. Greater than 75% of rabies victims die at home and these deaths are not included in the total deaths due to rabies. Up to 60% of rabies cases are in children, making rabies the seventh most important infectious disease in terms of years of life lost. Current vaccines regimens that are used to prevent rabies in humans are complicated and costly. In this report, we describe a novel mechanism by which to improve rabies vaccination by incorporating a membrane-anchored molecular adjuvant into the surface of the rabies virus particle. This new vaccine strategy significantly enhanced the speed and magnitude of the anti-rabies antibody responses and has the potential to improve the efficacy of currently used inactivated RABV-based vaccines.

## Introduction

Correlates of immunity for most human vaccines rely on antibodies for protection [1]. In the context of preventing human RABV infections, the induction of rapid and long-lasting serum VNAs is critical for protection because RABV vaccines are administered for both pre- and post-exposure settings (reviewed in [2, 3]). Strategies aimed at enhancing the speed, magnitude and longevity of vaccine-induced antibody titers is critical to improve vaccines against human rabies infection and/or to develop vaccines against other infectious diseases where an effective vaccine is lacking. One strategy to improve vaccine immunity is to target antigen to cells of the immune system. Typically, vaccine antigen is coupled to molecules specific for receptors on dendritic cells (DCs) and then inoculated with adjuvant [4–7]. Alternatively, targeting antigen directly to B cells improves the efficacy of antibody-based vaccines by increasing the speed [8] and magnitude of T cell-independent (TI) and T cell-dependent (TD) B cell responses [reviewed in [9]]. Fusing secreted antigen to CD180 [8] or C3d [reviewed in [9]] targets the antigen to cognate B cells, promoting rapid and potent antibody responses against viruses [10–15], bacteria [16], or synthetic antigens [17].

Our published data show that cell-free RABV particles migrate to the draining lymph node [18] and RABV vaccine strains directly target and activate primary murine and human B cells [19, 20]. Based on this, we hypothesized that exploiting the highly repetitive structure of proteins on the surface of RABV particles, and the natural tropism of attenuated RABV particles directly to B cells, would promote rapid and long-lasting antibodies responses. Specifically, we describe a novel RABV-based vaccine vector that displays both the antigen (RABV G) and molecular adjuvant (BAFF) on the surface of the same RABV particle to activate antigen-specific B cells.

BAFF is a molecule expressed mostly by cells of the innate immune system as well as by some T and B cells [reviewed in [21, 22]]. BAFF binds to the receptors B cell maturation antigen (BCMA), the transmembrane activator and calcium modulator and cyclophilin ligand interactor (TACI), and BAFF Receptor (BAFFR). These receptors are expressed on a wide range of differentiated B cells, including marginal zone B cells, B1 B cells, follicular B cells, or CD138^+^ antibody-secreting cells in secondary lymphoid organs. These receptors can also be detected on B cells at the site of infection, such as in the lungs during an influenza infection [23]. Due to the specificity of receptor expression on B cells, BAFF has the ability to modulate a wide range of B cell functions and enhance the efficacy of antibody-based vaccination, including: 1) mediating B cell survival and proliferation; 2) increasing protective IgG antibody titers [24]; 3) inducing and maintaining T and B cell responses, including antibody secreting cells, which are the most important effector B cell population in the context of protection against RABV infection; 4) sustaining antibody responses to influenza virus by maintaining antibody-secreting cells [23]; 5) enhancing antibody-mediated protection in models of other infectious diseases, including HIV, pneumococcus, malaria, Trypanosoma cruzi (Chagas disease) and RSV [25–29]; and, 6) BAFF potently augments B1 B cells to secrete IgM [29]. Importantly, BAFF influences B cell proliferation, differentiation and long-term survival of antiviral antibody secreting cells during recovery from alphaviral encephalomyelitis [30], suggesting BAFF may influence B cell responses in the CNS as well as in peripheral sites, which may help to improve vaccine-induced immunity against other neurotropic viruses, such as RABV.

We previously showed that expressing secreted BAFF, but not a proliferating inducing ligand (APRIL), from a recombinant RABV-based vaccine targets the extrafollicular pathway of B cell differentiation and improves rabies vaccinations [31, 32]. However, this approach requires viral gene expression *in-vivo* to produce BAFF, eliminating its potential as an inactivated vaccine. To circumvent this issue, and to target antigen-specific B cells directly, we incorporated membrane-anchored BAFF into the viral membrane. We show that membrane-anchored BAFF improves the speed and magnitude of vaccine-induced antibody response in live attenuated and inactivated RABV-based vaccines.

## Materials and methods

### Ethics statement

All animal work was reviewed and approved by the Institutional Animal Care and Use Committee (IACUC) of Jefferson Medical College, Thomas Jefferson University (Animal protocol #01838). Work was completed in accordance with international standards [Association for Assessment and Accreditation of Laboratory Animal Care (AAALAC)] and in compliance with Public Health Service Policy on Humane Care and Use of Laboratory Animals, The Guide for the Care and Use of Laboratory Animals of the National Institutes of Health (NIH).

### Construction and optimization of membrane-anchored molecular adjuvant

**Fig 1:**
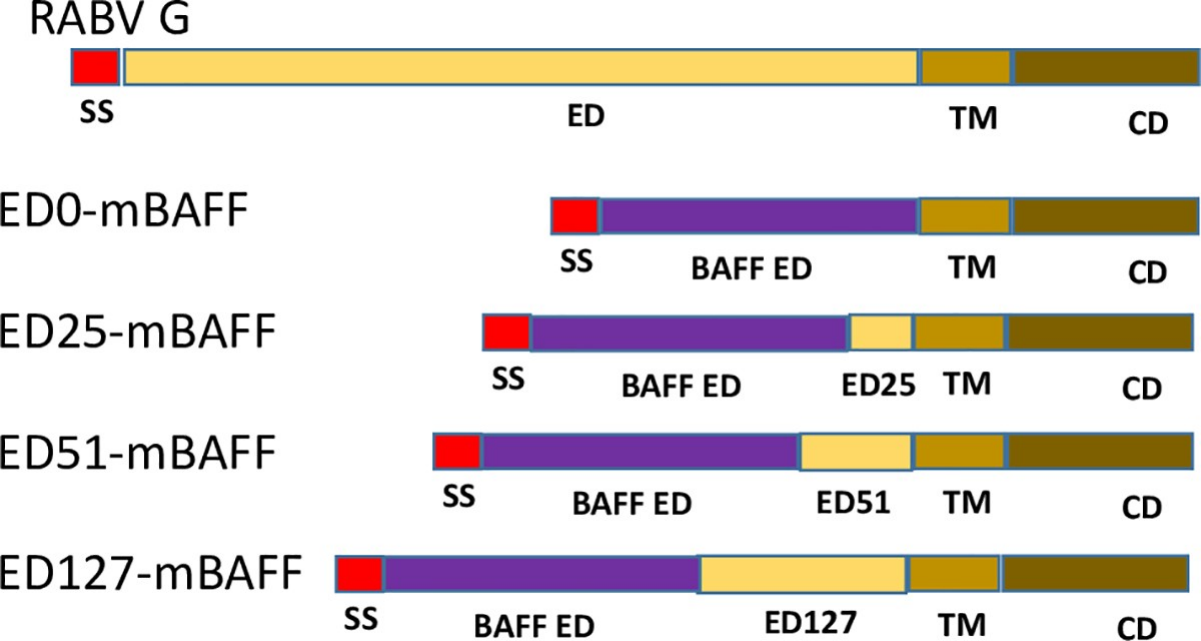
Construction of virus membrane-anchored murine BAFF. To construct a chimeric RABV G/BAFF fusion protein, the RABV G signal sequence and ED was replaced with an IL-2 signal sequence and the BAFF ED (ED0-mBAFF). Membrane-proximal 25, 51, or 127 amino acids of the RABV G ED were reintroduced into ED0-mBAFF, resulting in ED25-mBAFF, ED51-mBAFF and ED127-mBAFF, respectively.

Genes encoding viral membrane-anchored murine BAFF were synthesized by Genscript (Piscataway, NJ). The genes included (5’ to 3’): the restriction enzyme sites *EcoRI* and *BsiWI*, an IL-2 signal sequence, the soluble form of mouse BAFF (Accession number BC106841) fused in-frame with 0, 25, 51, or 127 membrane-proximal amino acids of the SAD-B19 RABV G ectodomain (ED), RABV G transmembrane domain (TM), RABV G cytoplasmic domain (CD), and *NheI* and *BamHI* restriction sites (Fig 1). The genes were cloned into expression plasmid pcDNA3.1(-) using the restriction sites *EcoRI* and *BamHI*, resulting in pcDNA-ED0-mBAFF, pcDNA-ED25-mBAFF, pcDNA-ED51-mBAFF or pcDNA-ED127-mBAFF. BSR cells were transfected with 2 μg of each plasmid. Three days later, the cells were collected and analyzed for surface expression of BAFF using rat anti-mouse BAFF monoclonal antibody (R&D Systems; Clone #121808). Samples were fixed in 4% paraformaldehyde and analyzed on a BD LSRFortessa cell analyzer. Data were analyzed using FlowJo (FlowJo, LLC.) and Prism 5 (Graphpad). An unpaired, two-tailed Student’s t test was used to compare mean fluorescent intensity (MFI) between experimental transfection with mock-transfected cells (*p<0.05; **p<0.01; N = 3 from 2 independent experiments completed in duplicate).

### Vaccine construction, recovery and characterization

#### Construction and recovery of RABV-ED51-mBAFF

RABV is a molecular clone of the vaccine strain of rabies virus, SAD-B19 [33–35]. To construct a RABV-based vaccine vector expressing virus membrane anchored murine BAFF, ED51-mBAFF was digested from pcDNA-ED51-mBAFF using the restriction enzymes *BsiWI* and *NheI* and then inserted into pRABV also digested with *BsiWI* and *NheI*, resulting in pRABV-ED51-mBAFF. Infectious virus was recovered as described previously [20] and named RABV-ED51-mBAFF.

#### Western blot analyses of sucrose-purified RABV-ED51-mBAFF particles

BSR cells were seeded at 5 x 10^6^ cells in t75 tissue culture flask in Dulbecco’s Modification of Eagles media (DMEM) containing 5% heat-inactivated fetal bovine serum (FBS) (Gibco)/1% Penicillin/Streptomycin (PS) (Corning) and infected with RABV or RABV-ED51-mBAFF at a multiplicity of infection (MOI) of 0.1 and incubated for 3 days at 37°c and 5% CO_2_. Supernatants were harvested and clarified of cell debris by centrifugation (5 min., 3,000g, room temperature). Viral pellets were collected by ultra-centrifugation for 1 hour, 24k rpm at 4°C (Beckman SW-28 rotor) over 20% sucrose/PBS cushion. Purified virus was reconstituted in 200ul PBS and incubated overnight at 4°C. Purified virus was reduced and denatured, separated by SDS-PAGE, transferred to polyvinylidene fluoride membrane, and blocked with 5% non-fat milk (LabScientific) in PBS overnight. Membrane was probed for one hour with polyclonal goat IgG anti-murine BAFF primary antibody (AF2106; R&D Systems) at a dilution of 1:2,000 in PBS-0.05% Tween-20 (PBS-T), washed 3 times with PBS-T, then incubated for one hour with donkey anti-goat IgG horseradish peroxidase-conjugated secondary antibody (Jackson Immuno) diluted 1:30,000 in PBS-T. The membrane was developed using ECL Western blotting substrate (Pierce). Bots were analyzed using Flurochem M System. Protein deglycosylation was completed on sucrose purified RABV-ED51-mBAFF as described by the manufacturer (New England Biolabs, Protein Deglycosylation Mix II; P6044) and then analyzed by Western blot analysis as just described.

#### One-step and multi-cycle growth curves

BSR cells were seeded at 5 x 10^5^ cells/well in a 6 well plate in DMEM containing 5% heat-inactivated FBS/1% PS. Wells were infected 24 hours later with a MOI of 0.01 or 5 for multi-cycle or one-step growth curves, respectively. One-hour post-infection, cells were washed 3 times with PBS to remove excess virus and then incubated at 37°C and 5% CO_2_. 100 μl aliquots of tissue culture supernatants were harvested at 24, 48, 72 and 96 hours post-infection. Wells were refed DMEM after each harvest. Titers of harvested supernatants were determined on BSR cells in duplicate after 48 hour incubation at 37°C and 5% CO_2_ as described [36, 37]).

### *Ex Vivo* primary B cell survival and activation

#### Primary murine B cell survival

Spleens were harvested from naïve 8–10 week old female C57BL/J6 mice (Jackson) and single-cell suspensions prepared [38–40]. Red blood cells were lysed using ACK lysis buffer (A1049201; Thermofisher), filtered by 70 micron filter, and seeded at a density of 5 x 10^6^ /ml in splenocyte media (RPMI 1640 containing 10% FBS, 50 μM beta-mercaptoethanol, 100Ul/mL PS, and 100 mM HEPES). Cells were infected with a MOI of 5 with sucrose purified RABV, RABV-ED51-mBAFF, or RABV-ED51-mBAFF pre-treated for 2 hours at 37°C with 5μg/ml an antibody [20] (Sandy-2; Adipogen) that neutralizes BAFF function by inhibiting mouse BAFF binding to its receptors. Two days later, cells were harvested and plated at 10^6^ cells/well of a 96-well plate, pelleted at 300 x g, washed in FACS Buffer (PBS containing 2% FBS). Cells were incubated with Fixable Live/Dead-DAPI (Thermofisher), washed with FACS Buffer and incubated with CD16/32 FcBlock (BD Biosciences). Cells were stained with 0.2 μg/ml anti-B220-PE (Invitrogen, 12-0452-82) for 30 minutes. Cells were fixed in 3% paraformaldehyde (Affimetrex) for 30 minutes, washed, and resuspended in FACS buffer and analyzed using BD Fortessa flow cytometer. Data was analyzed using FlowJo Software and significance was calculated using unpaired, two-tailed Student’s t test in Prism 6 (Graphpad) software. To compare two groups of data, an unpaired two-tailed Student’s t test was used (*p≤0.05; **p≤ 0.01; N = 2 completed in duplicate).

#### Primary murine B cell activation

Spleens were harvested as described above and cell suspensions were infected at a MOI of 5 with RABV, RABV-ED51-mBAFF or equivalent volume of PBS, and incubated for 2 days 37°C and 5% CO_2_. Cells were harvested and plated at 10^6^ cells/well of a 96-well plate, pelleted at 300 x g, washed in FACS Buffer (PBS containing 2% FBS). Cells were incubated with Fixable Live/Dead-Aqua (Thermofisher), washed with FACS Buffer and incubated with CD16/32 FcBlock (BD Biosciences). Cells were stained with surface antibody mixture, including (0.2 ug/ml each) anti-B220-PerCP (Clone RA6B2; BD Biosciences), anti-CD40-APC (Clone 1C10; eBiosciences), anti-CD69-V450 (Clone 41:2F3; BD Biosciences), and anti-MHC-II-Alexa Fluor 700 (Clone M5/11415.2; BD Biosciences) for 30 minutes. Cells were fixed in 3% paraformaldehyde (Affimetrex) for 30 minutes, washed, and permeabilized using BD Perm/Wash (554723; BD Biosciences) for anti-Rabies-N-FITC (FujiRebio) intracellular staining. Cells were suspended in FACS buffer and analyzed using LSRII flow cytometer. Data was analyzed using FlowJo Software. To compare two groups of data, an unpaired, two-tailed Student’s t test was use (*p≤0.05; **p≤0.01; ***p≤0.001; N = 3 completed in duplicate).

#### Mouse immunogenicity studies: Evaluation of antibody responses by ELISA and Rapid Fluorescent Foci Inhibition Test (RFFIT)

Groups of 8–10 weeks old C57BL/J6 female mice (Jackson) were immunized intramuscularly (i.m) via gastrocnemius with 100 μl (50 μl/leg) of live or inactivated RABV or RABV-ED51-mBAFF as indicated in the figures. Inactivated RABV and inactivated RABV-ED51-mBAFF were prepared as follows: virus stocks were grown in OptiPRO Serum Free Media (Gibco) [4mM L-Glutamine, 1% PS], harvested, and cell debris was removed using Corning 0.45μm filter (430516; Corning). β-Propiolactone (BPL; P5648; Sigma) was added to viral supernatants (final concentration 0.05% BPL), and incubated overnight at 4ᵒC. Treated supernatants were purified using ultracentrifugation. Viral inactivation was confirmed by viral titer [37]. Total protein concentrations were quantified by BCA Protein Assay Kit as described by the manufacturer (Pierce). Blood from immunized mice was collected via retro-orbital at 5, 7, 10 days post-immunization. RABV G-specific IgM and IgG (and subclasses) antibody levels were determined by ELISA as described previously [36–39]. VNA titers were determined by RFFIT as described previously [36, 37, 40].

## Results

### Fifty-one (51) membrane-proximal amino acids of the RABV G ED are required for surface expression of virus membrane-anchored BAFF

**Fig 2:**
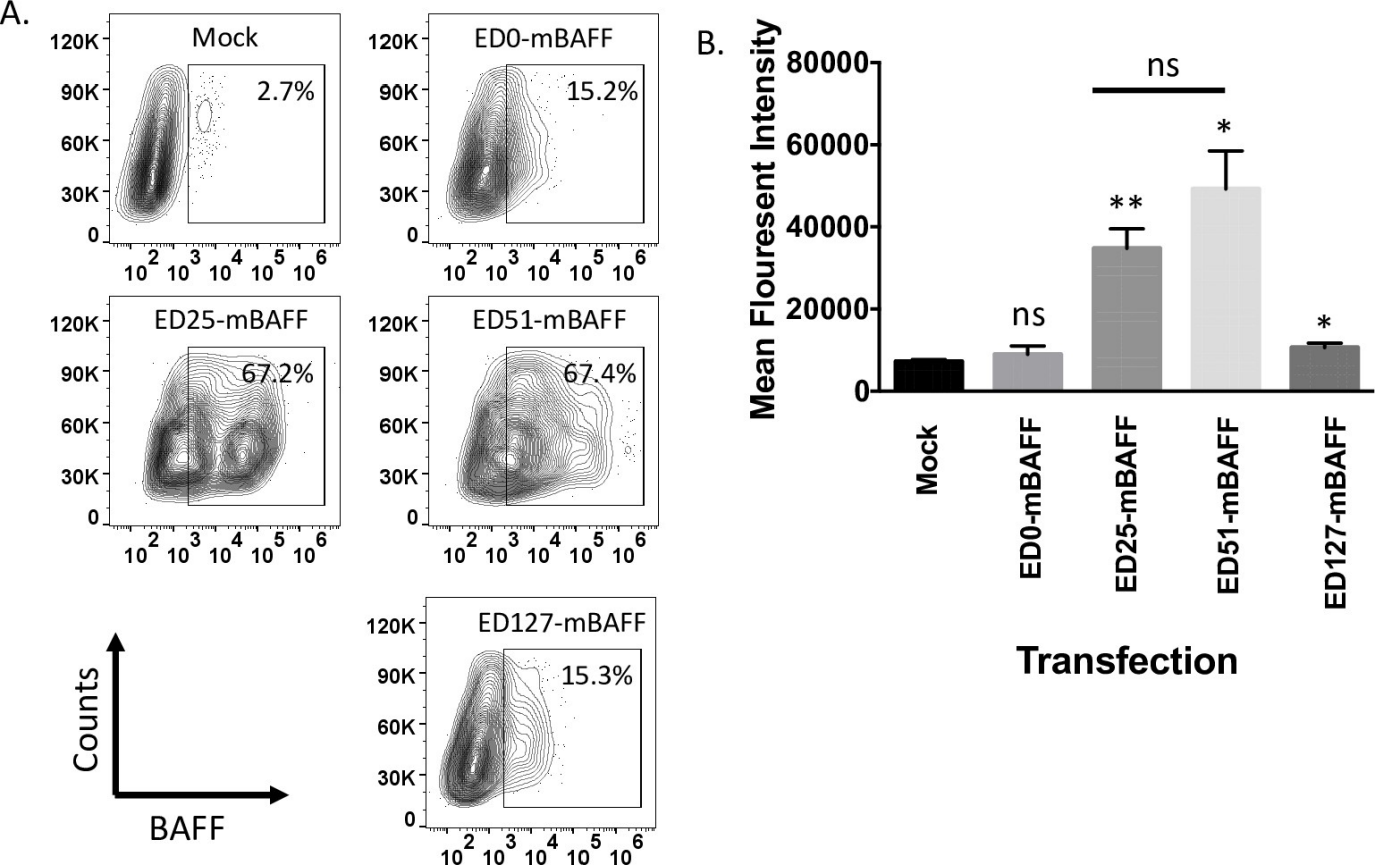
Twenty five or 51 amino acids of the membrane-proximal RABV G ED support the transport of BAFF to the surface of cells. BSR cells were transfected with pcDNA-ED0-mBAFF, pcDNA-ED25-mBAFF, pcDNA-ED51-mBAFF or pcDNA-ED127-mBAFF and three days later the cells were collected and fixed with 3% paraformaldehyde. Mock (PBS)-treated cells served as negative control cells. Cells were stained with mouse BAFF antibody conjugated to PE. Immunostained cells were analyzed by FACs analysis for BAFF surface expression. (A) Representative gating strategy to determine BAFF expression on the surface of BSR cells. (B) The MFI of surface expression of BAFF is summarized. To compare BAFF expression levels from each chimeric BAFF/RABV G fusion protein to mock-treated cells, an unpaired two-tailed Student’s t test was used (*p≤0.05; **p≤0.01; ns = not significant; N = 3 from 2 independent experiments completed in duplicate).

In this project, we aimed to exploit the natural ability for attenuated RABV-based vaccine strains to target B cells for infection and activation [19, 20, 41] by further directing RABV particles to B cells. To that end, we cloned and recovered a recombinant RABV-based vaccine expressing the wild-type RABV G (antigen) as well as virus membrane-anchored BAFF (adjuvant). The membrane-anchored BAFF in this first experiment consisted of an IL-2 signal sequence fused in-frame with the ectodomain of murine BAFF (mBAFF) and the RABV G TM and CD (RABV-mBAFF). However, this chimeric BAFF/RABV G fusion protein was not trafficked to the cell surface or incorporated into RABV particles. It was previously shown that some foreign proteins require additional membrane-proximal amino acids of the RABV G ED to support surface expression and incorporation into RABV particles [42]. To determine whether membrane-proximal amino acids of the RABV G ED improved surface expression of BAFF, we cloned a series of expression plasmids encoding for an IL-2 signal sequence, soluble murine BAFF fused in-frame with 0, 25, 51, or 127 membrane-proximal amino acids of the RABV G ED, RABV G TM, and RABV G CD, resulting in pcDNA-ED0-mBAFF, pcDNA-ED25-mBAFF, pcDNA-ED51-mBAFF or pcDNA-ED127-mBAFF (Fig 1). BSR cells were transfected with each plasmid and the expression of BAFF on the surface of cells was measured by FACS analysis. Representative gating strategies used to identify BAFF surface expression are shown in Fig 2A and a summary of the data is provided in Fig 2B. BAFF expression on the surface of cells transfected with pcDNA-ED0-mBAFF was not significantly different from mock-transfected cells. This is consistent with our finding above that BAFF was not trafficked to the cell surface when infected with RABV-mBAFF. Significant, but low levels of surface expression of BAFF were detected on the surface of BSR cells transfected pcDNA-ED127-mBAFF. High levels of BAFF were detected on the surface of BSR cells transfected with pcDNA-ED25-mBAFF or pcDNA-ED51-mBAFF. Together, these data indicate that the addition of 25 or 51 amino acids of the membrane-proximal RABV G ED supports the transport of BAFF through the endoplasmic reticulum, Golgi apparatus, and to the cell surface. Based on previous findings that ED51 supported efficient cell surface expression of an unrelated proteins [42], we used the gene encoding for the ED51-mBAFF fusion protein in subsequent experiments.

### ED51-mBAFF is efficiently incorporated into RABV-ED51-mBAFF particles while not influencing virus growth kinetics

**Fig 3:**
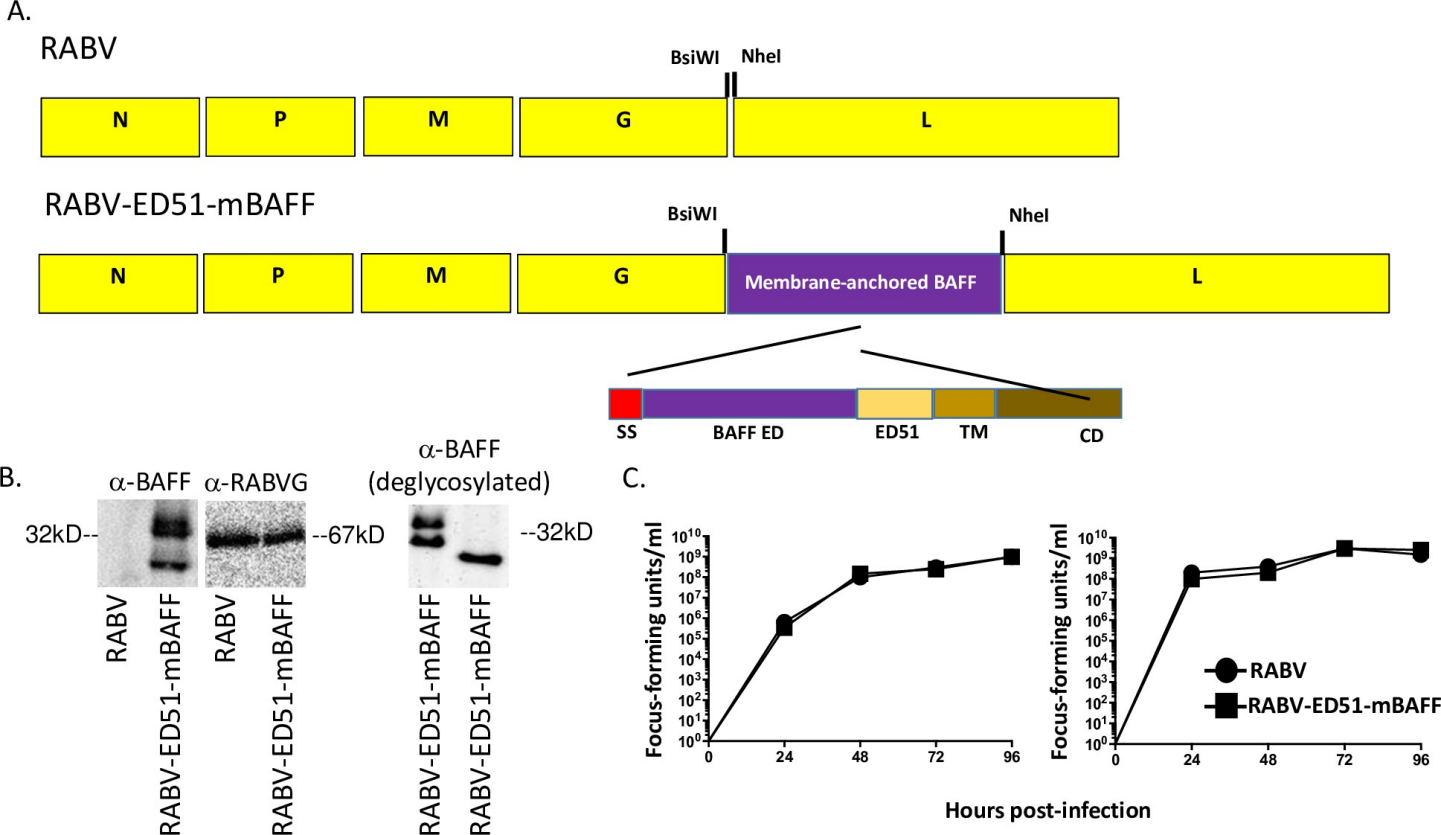
Construction and characterization of a recombinant RABV-based vector displaying virus membrane-anchored BAFF (RABV-ED51-mBAFF). (A) RABV is a molecular clone of the attenuated vaccine strain of RABV, SAD-B19. The gene encoding ED51-mBAFF from pcDNA-ED51-mBAFF (Fig 1) was digested with *BsiWI* and *NheI* and then ligated into pRABV previously digested with *BsiWI* and *NheI*, resulting in pRABV-ED51-mBAFF. Infectious virus was recovered and named RABV-ED51-mBAFF. (B) To analyze incorporation of ED51-mBAFF into RABV-ED51-mBAFF virions, sucrose-purified RABV or RABV-ED51-mBAFF particles were separated by SDS-PAGE and transferred to a nitrocellulose membrane. Blots were probed with either an anti-BAFF antibody (left panel) or anti-RABV G antibody (right panel). (C) BSR cells were infected with RABV or RABV-ED51-mBAFF at a MOI of 5 (one-step growth curve, right panel) or 0.01 (multi-cycle growth curve, left panel). Aliquots of tissue culture supernatants were collected at various times post-infection and viral titers were determined in duplicate.

The results described above indicate that the inclusion of 51 membrane-proximal amino acids of the RABV G ED would support the incorporation of BAFF into the membrane of RABV particles. To test this hypothesis, a recombinant RABV-based vector was cloned and recovered that expresses ED51-mBAFF (RABV-ED51-mBAFF) (Fig 3A). Western blot analysis of sucrose-purified RABV or RABV-ED51-mBAFF showed that ED51-mBAFF was incorporated into RABV-ED51-mBAFF particles (Fig 3B). Of note, the two bands that were detected with the anti-BAFF antibody were reduced to a single band when deglycosylated prior to a Western blot analysis (Fig 3B). This is consistent with the finding that BAFF is a peptide glycoprotein [43]. One-step (Fig 3C, left) and multi-cycle (Fig 3C, right) growth curves showed that RABV-ED51-mBAFF grew to titers similar as RABV in BSR cells. Similar growth kinetics indicate that the insertion of this foreign gene into the RABV genome, or the incorporation of the chimeric protein into the virus particle, did not affect the ability for RABV-ED51-mBAFF to infect, replicate or spread from cell-to-cell *in-vitro*.

### ED51-mBAFF is functional and increases survival of RABV-ED51-mBAFF-treated primary murine B cells

**Fig 4:**
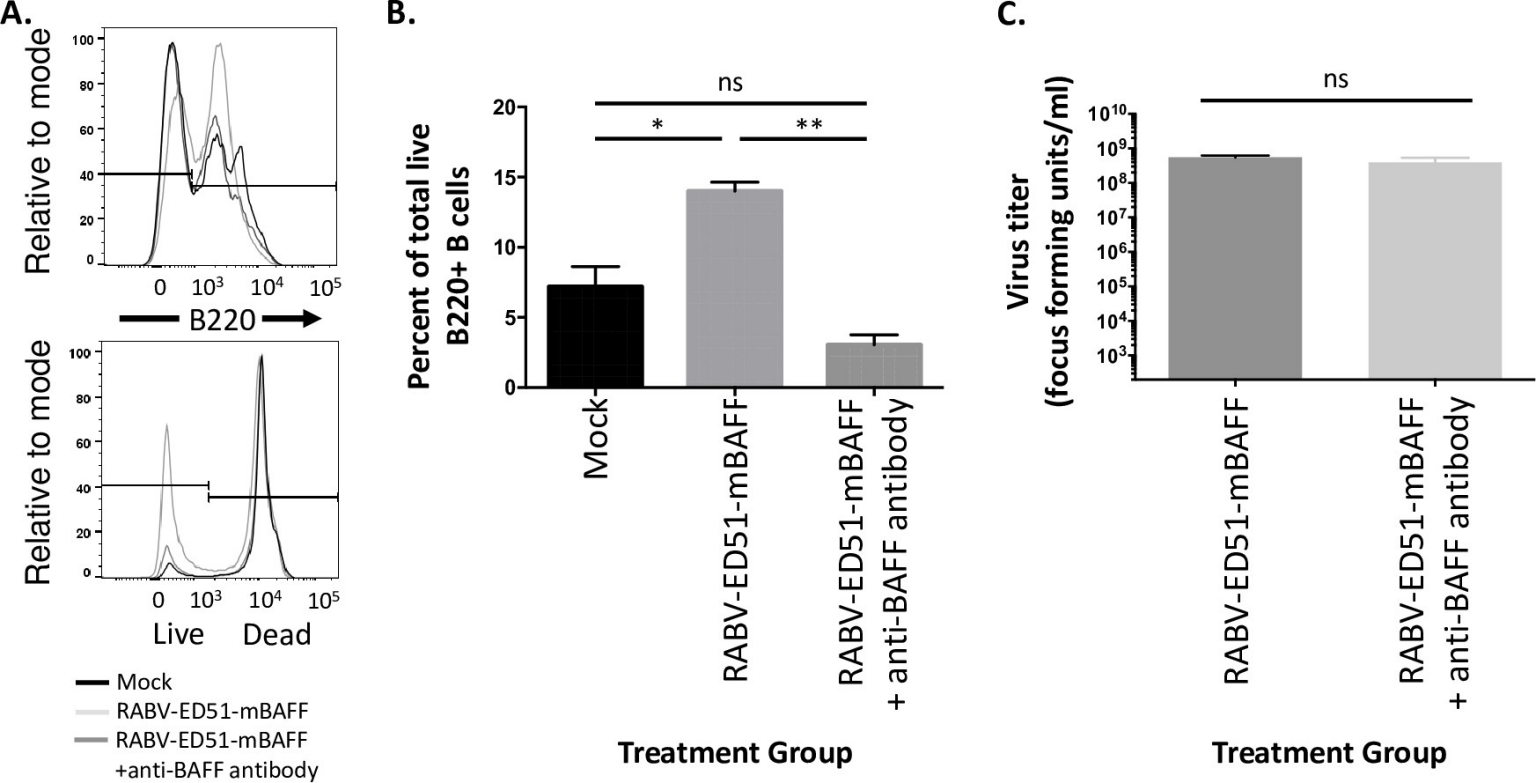
Virus membrane-anchored ED51-mBAFF is functional. Naïve primary murine splenocytes were infected with a MOI of 5 with sucrose-purified RABV, RABV-ED51-mBAFF, or RABV-ED51-mBAFF pre-treated with a neutralizing anti-BAFF antibody. Splenocytes treated with media alone (mock-infected) served as a negative control. No additional mitogens were added to the culture to maintain the B cells and accessory splenocytes in a resting state similar to that in which they would exist *in-vivo* at the time of initial immunization. Two days later, cells were collected and analyzed for B cell survival by staining cells with anti-B220-PE antibody and Fixable Live/Dead-DAPI stain followed by FACS analyses. (A) Gating strategy for the presence of live B220^+^ cells. (B) Summary graph showing the percent of live B220^+^ cells. (C) BSR cells (a derivative of baby hamster kidney cells) were infected with a MOI of 5 with sucrose-purified RABV-ED51-mBAFF or RABV-ED51-mBAFF pre-treated with a neutralizing anti-BAFF antibody. Two days later, supernatants were collected and titered on BSR cells. To compare two groups of data, an unpaired two-tailed Student’s t test was used (*p≤0.05; **p≤0.01; ns = not significant; N = 2 completed in duplicate).

To confirm that the viral membrane-anchored ED51-mBAFF was functional, primary murine splenocytes treated with RABV-ED51-mBAFF showed a significant, three-fold increase in B cell survivorship compared to cells treated with RABV (Fig 4A and 4B). Pre-treating RABV-ED51-mBAFF with an antibody that neutralizes BAFF function (Clone Sandy-2) [44] reduced B cell survival to levels detected in mock-treated cells, demonstrating the increase in B cell survivorship was due to BAFF on the surface of the viral particle. This is consistent with the findings by others that endogenously produced BAFF improves B cell survival [43]. Of note pre-treating RABV-ED51-BAFF with the anti-BAFF neutralizing antibody did not influence the ability for the virus to infect BSR cells (Fig 4C), indicating that the antibody is not hindering B cell infection and activation via steric hinderance. Together, the chimeric ED51-mBAFF protein, which is incorporated into the virus membrane, is biologically active.

### RABV-ED51-mBAFF targets primary murine B cells for infection and activation *ex-vivo*

**Fig 5:**
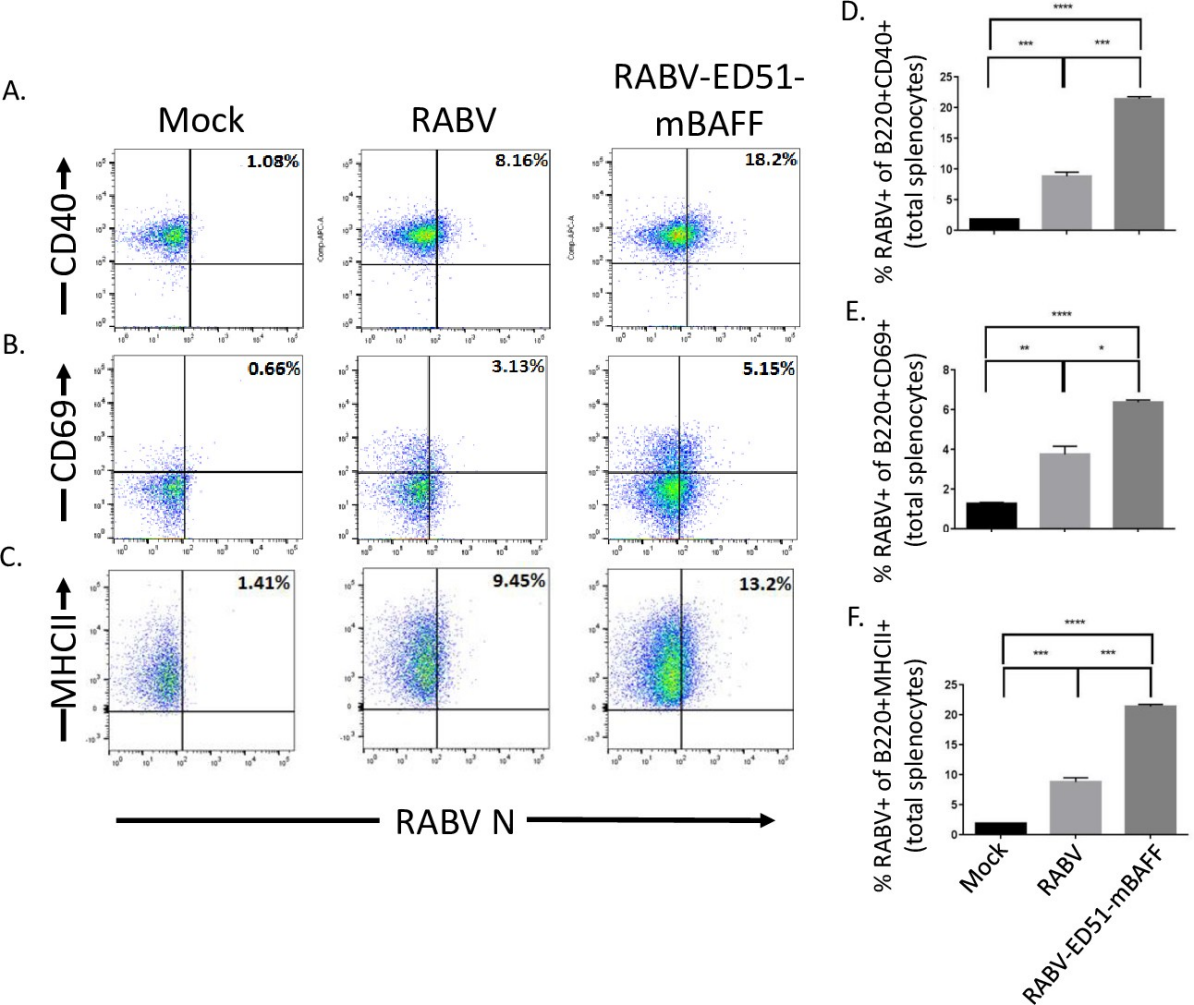
RABV-ED51-mBAFF activates primary murine B cells more effectively than RABV *ex-vivo*. Naïve primary murine splenocytes were infected at a MOI of 5 with RABV or RABV-ED51-mBAFF, or treated with medium alone (mock infected) for two days *ex-vivo*. No additional mitogens were added to the culture to avoid expressing activation molecules that could enhance sensitivity to RABV infection and activation. The cells were immunostained for cell-surface expression of B220 and the activation markers CD40, CD69, and MHCII, as well as immunostained internally for the presence of RABV N, which is indicative of infection. Representative gating strategy are shown for B220^+^ B cells gated on RABV N^+^ and CD40 (A), CD69 (B) or MHCII (C). The percentage of RABVN^+^CD40^+^ B cells (D), RABVN^+^CD69^+^ B cells (E) or RABVN^+^MHCII^+^ B cells (F) are indicated. To compare two groups of data, an unpaired, two-tailed Student’s t test was use (*p≤0.05; **p≤0.01; ***p≤0.001; ****p≤0.0001; N = 3 completed in duplicate).

Based on our previous findings that live attenuated RABV vaccine strains activate primary murine and human B cells *ex-vivo* [19, 20], we hypothesized that a recombinant RABV-based vaccine that incorporates membrane-anchored BAFF into the virus particle would promote B cell infection and enhanced B cell activation *ex-vivo*. Naïve primary murine splenocytes infected RABV-ED51-mBAFF showed a significant increase in the percentage of RABV N^+^ B220^+^ B cells that express the activation markers CD40 (Fig 5A and 5D), CD69 (Fig 5B and 5E) and MHCII (Fig 5C and 5F) compared with cells treated with RABV. Only background levels of RABV N^+^ B220^+^ cells were detected in mock-treated splenocytes. Together, these data confirm: i.) data from Fig 4 showing that ED51-mBAFF is functional, ii.) our previous reports showing that attenuated RABV-based strains activate naïve primary murine B cells [19, 20], and iii) that expressing virus membrane-anchored BAFF from RABV-ED51-mBAFF enhances B cell activation in RABV-targeted B cells.

### Live attenuated RABV-ED51-mBAFF induces rapid antibody responses in mice

**Fig 6:**
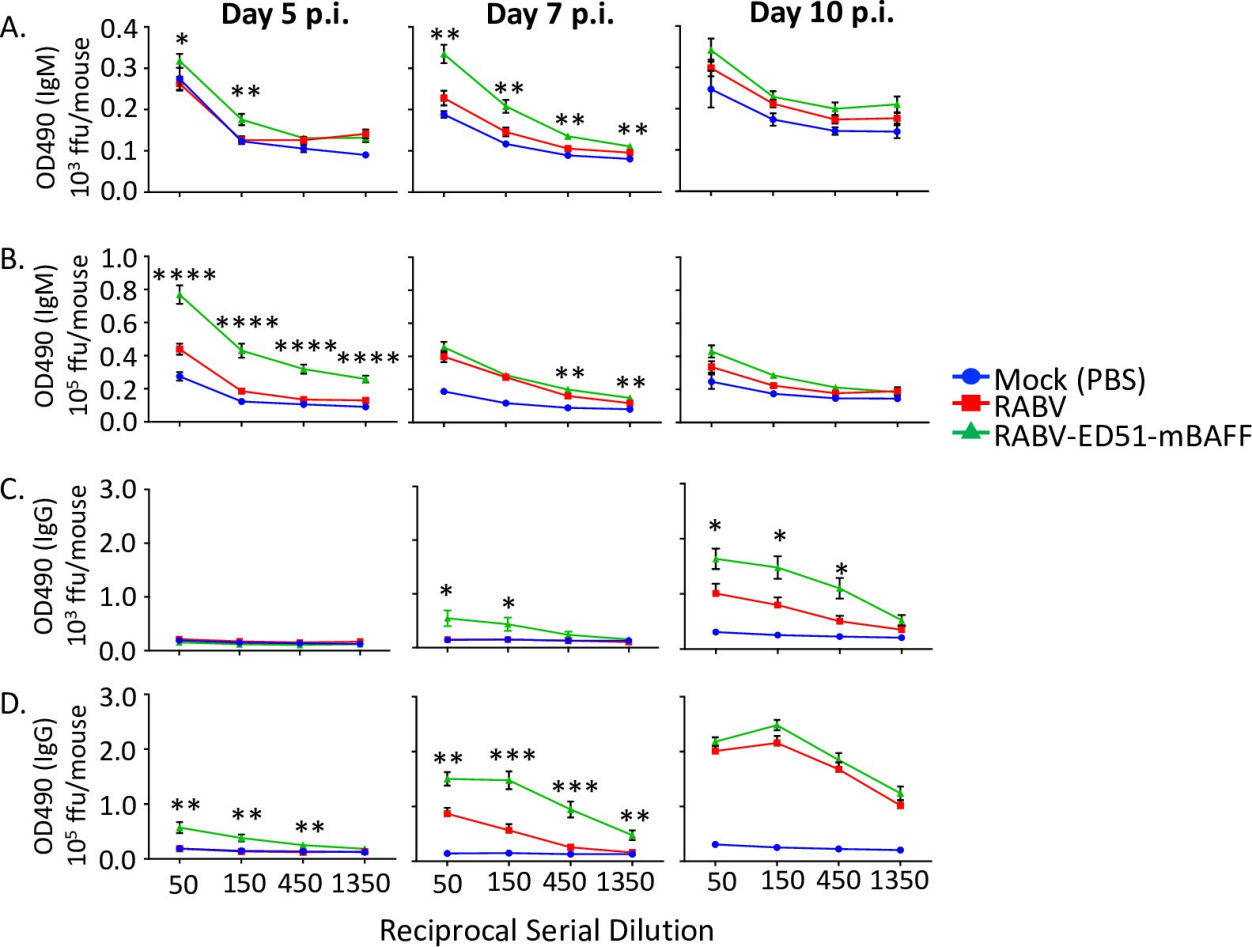
100-fold less RABV-ED51-mBAFF is needed to induce comparable immunity in mice immunized with RABV. C57BL/6J mice were immunized i.m. with 10^3^ or 10^5^ ffu/mouse of RABV or RABV-ED51-mBAFF, or PBS as a negative control. Blood was collected on days 5, 7 and 10 post-immunization as a source of serum. Four three-fold serial dilutions of sera were analyzed by ELISA to determine anti-RABV G IgM (A and B) or IgG (C and D) antibody titers and presented as OD_490_ of the reciprocal serial dilution. For comparison, sera from PBS-immunized mice were tested in parallel. Statistical difference in antibody titers by ELISA between RABV- and RABV-ED51-mBAFF-immunized mice was determined using an unpaired, two-tailed t test and data is presented as the mean ± SEM. *p≤0.05; **p ≤ 0.01; ***p≤0.001; ****p≤0.0001; N = 10 mice/group). (ffu = focus forming units; OD = optical density).

The above data indicate that RABV-ED51-mBAFF activates naïve primary murine B cells more effectively than does RABV *ex-vivo*, suggesting that RABV-ED51-mBAFF might improve B cell responses *in-vivo* compared to RABV. Mice immunized with 10^3^ (Fig 6A) or 10^5^ (Fig 6B) ffu/mouse of RABV-ED51-mBAFF showed significantly higher RABV G-specific IgM antibody responses as early as 5 days post-immunization with as little as 10^3^ ffu/mouse of virus compared to mice immunized with the same dose of RABV. In addition, immunization with 10^3^ or 10^5^ ffu/mouse of RABV-ED51-mBAFF induced significantly higher and faster RABV G-specific IgG antibody responses compared to RABV at almost all time points tested (Fig 6C and 6D). Of note, mice immunized with only 10^3^ ffu/mouse with RABV-ED51-mBAFF showed similar antibody kinetics compared to mice immunized with 10^5^ ffu/mouse with RABV indicating that 100-fold less RABV-ED51-mBAFF is needed to induce similar anti-RABV G antibody responses as the parental virus, RABV.

### Antibodies produced by RABV-ED51-mBAFF are neutralizing and biased towards a Th1-type response

**Fig 7:**
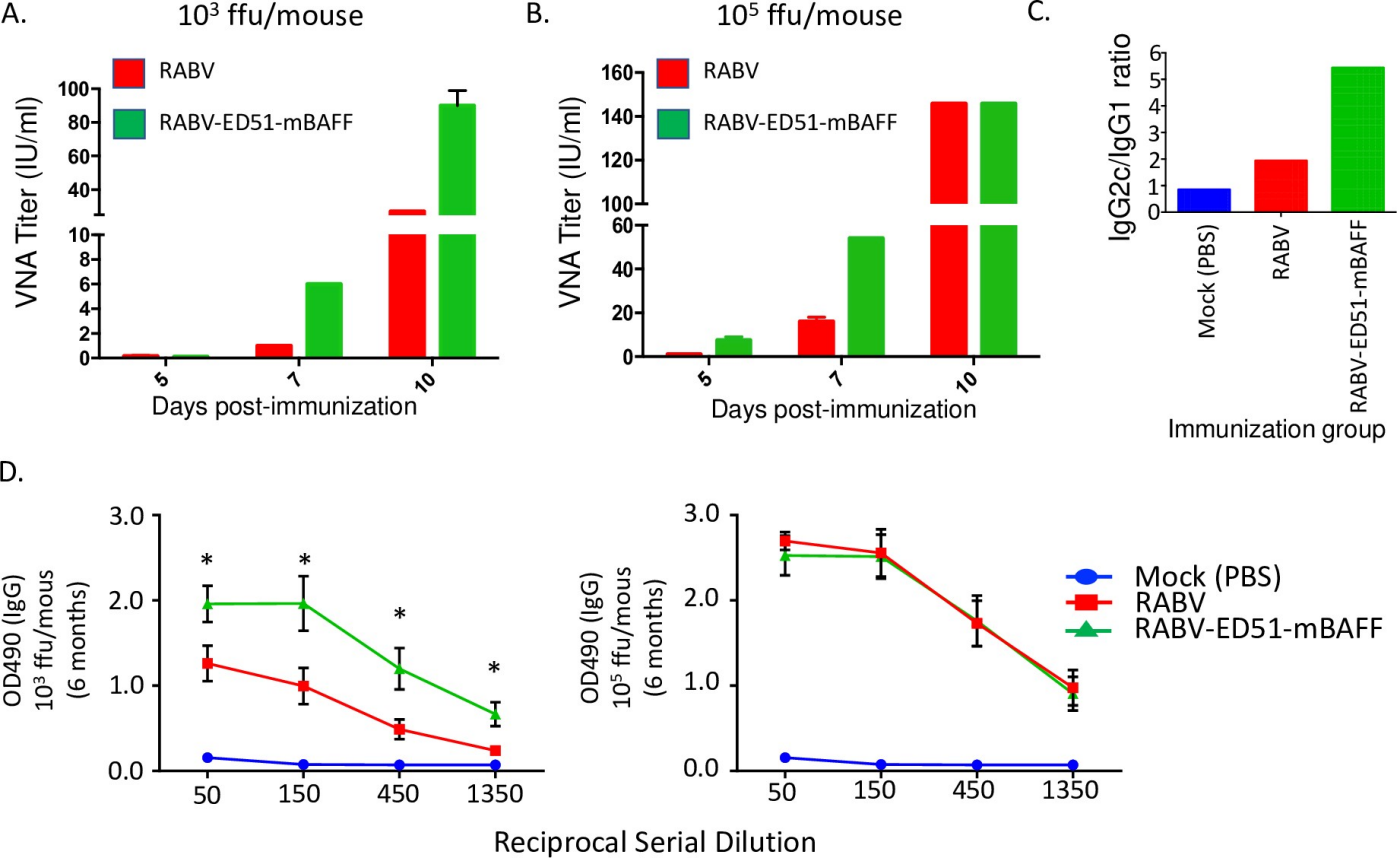
RABV-ED51-mBAFF induces potent VNA titers and a Th1-biased antibody response, which does not adversely affect long term immunity. Sera from mice immunized with 10^3^ (A) or 10^5^ (B) ffu/mouse of RABV-ED51-mBAFF or RABV were pooled and analyzed for VNA titers using RFFIT. Data represents sera from 5 mice per group pooled in two assays (N = 10/group) and completed in duplicate. International Units (IU)/ml were determined by comparing results to a WHO standard. Serum from mice immunized with 10^5^ ffu/mouse on day 14 post-immunization were analyzed by ELISA for anti-RABV G IgG1 and IgG2c titers. (C) The ratio of IgG2c/IgG1 is summarized from N = 10 mice per group. (D) Serum from mice immunized with 10^3^ (left) or 10^5^ ffu/mouse (right) 6 months post-immunization were analyzed for total anti-RABV G IgG titers. Statistical difference in antibody titers by ELISA between RABV- and RABV-ED51-mBAFF-immunized mice was determined using an unpaired, two-tailed t test and data is presented as the mean ± SEM. *p≤0.05 (N = 10).

VNAs directed against the single viral transmembrane glycoprotein are the primary correlate of immunity to protect against rabies infections. Consistent with the antibody titers measured by ELISA in Fig 6, VNA titers were higher in mice immunized with either 10^3^ (Fig 7A) or 10^5^ (Fig 7B) ffu/mouse of RABV-ED51-mBAFF compared with mice immunized with RABV. VNA titers almost 10-fold higher than the level suggestive of a satisfactory immunization were detected in mice immunized with 10^5^ ffu/mouse as early as 5 days post-immunization. In addition to the magnitude of vaccine-induced antibody responses, a vaccine that elicits potent Th1-type antibody responses, exemplified by an enhancement in the ratio of vaccine-induced IgG2c/IgG1 antibodies, might be beneficial in post-exposure settings when infection has already occurred [37]. RABV-ED51-mBAFF induced a highly Th1-biased antibody response as demonstrated by an IgG2c/IgG1 ratio of about 5. Together, the incorporation of BAFF into the membrane of RABV particles not only increases the magnitude and speed of the VNA titers, but also promotes a Th1-type antibody response. Antibody titers were consistent between mice immunized with RABV-ED51-mBAFF and RABV six months post-immunization, indicating altering early events in B cell activation do not adversely affect the ability for RABV-ED51-mBAFF to induce longer lasting immunity (Fig 7D).

### Inactivated RABV-ED51-mBAFF induces rapid antibody responses in mice

**Fig 8:**
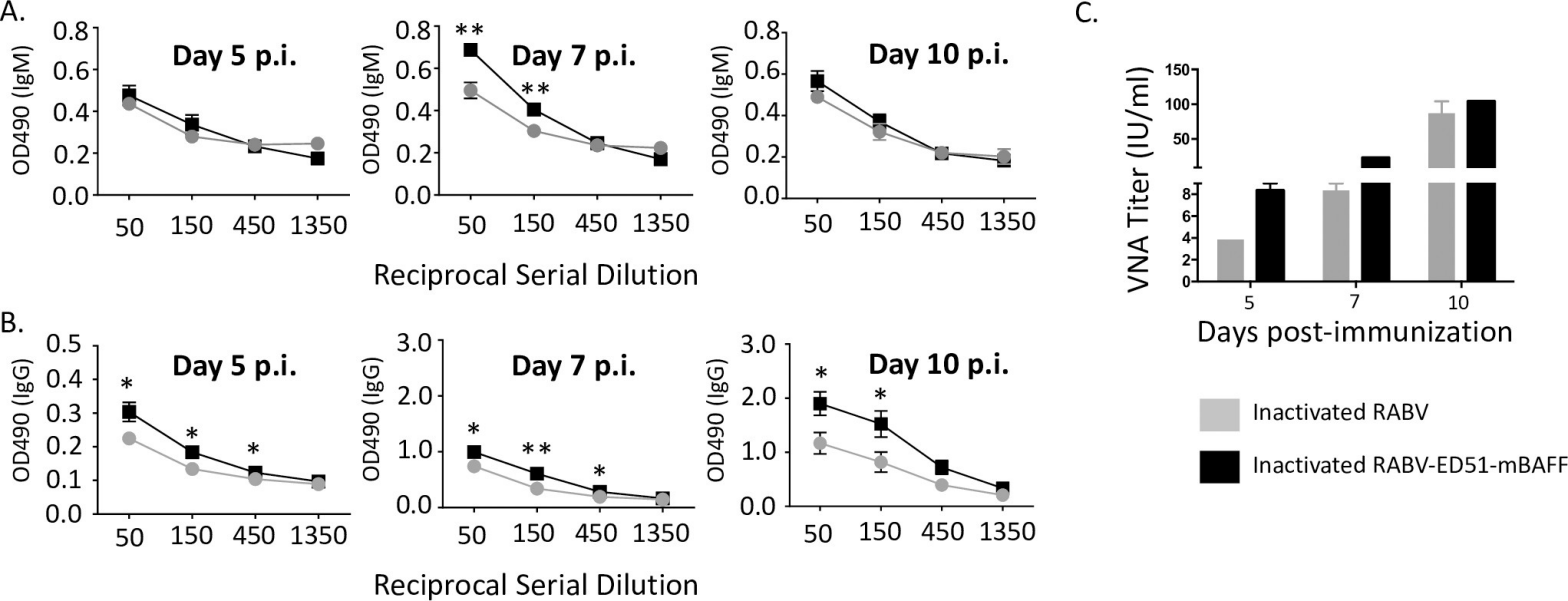
Inactivated RABV-ED51-mBAFF induces significantly faster and higher antibody responses in mice compared with inactivated RABV. C57BL/6 mice were immunized i.m. with 10 ug/mouse inactivated RABV or inactivated RABV-ED51-mBAFF and blood was collected on days 5, 7 and 10 post immunization. Four three-fold serial dilutions of sera were analyzed by ELISA to determine anti-RABV G IgM (A) or IgG (B) antibody titers and presented as OD_490_ of the reciprocal serial dilution. Statistical difference in antibody titers by ELISA between two groups of data was determined using an unpaired, two-tailed t test and data is presented at the mean ± SEM. (*p≤0.05; **p ≤ 0.01; ***p≤0.001; ****≤0.0001; N  =  8 mice per group from two independent experiments; OD  =  optical density). C) To confirm the antibodies were neutralizing and to determine whether lower doses of vaccine effectively improved immunity, a separate group of C57BL/6 mice were immunized with 250 ng/mouse with inactivated RABV or inactivated RABV-ED51-mBAFF. Blood was collected from immunized mice, pooled, and then tested by RFFIT for VNA titers (N = 5 mice per group in duplicate).

Because ED51-mBAFF is embedded into the viral membrane during propagation in tissue culture and viral genome replication is not required to produce BAFF *in-vivo*, we hypothesized that RABV-ED51-mBAFF would induce potent immunity as an inactivated vaccine. Mice immunized with 10 ug/mouse of inactivated RABV-ED51-mBAFF induced a slightly but significantly faster and higher anti-RABV G IgM (Fig 8A) and IgG (Fig 8B) antibody responses compared with mice immunized with equal doses of inactivated RABV. To determine whether lower doses of inactivated RABV-ED51-mBAFF improved vaccine-induced immunity compared with lower doses of inactivated RABV, mice were immunized with 250 ng/mouse of inactivated RABV-ED51-mBAFF or inactivated RABV. As shown in Fig 8C, VNA titers in mice immunized with inactivated RABV-ED51-mBAFF were almost 20-fold higher than the suggested VNA titers indicative of a satisfactory immunization (>0.5 IU/ml) 5 days post-immunization. The antibody responses induced by inactivated RABV-ED51-mBAFF exemplify the potential of incorporating membrane-anchored molecular adjuvants into the surface of an inactivated viral particle to improve vaccine-induced B cell responses.

## Discussion

B cells are strongly activated by highly structured, membrane-anchored antigen, such as the case with the viral glycoprotein displayed on the surface of RABV particles. In this project, we exploited the highly repetitive structure of RABV surface proteins to display both antigen and molecular adjuvant on the surface of the same virus particle. The molecular adjuvant, BAFF, was expressed on the virus surface as a chimeric RABV G/BAFF fusion protein. Virus membrane-anchored BAFF was shown to be functional, as demonstrated by the ability for RABV-ED51-mBAFF particles to prolong primary murine B cell survival compared with RABV alone. B cell survival was reduced by pre-treating RABV-ED51-mBAFF with a neutralizing anti-BAFF antibody. RABV-ED51-mBAFF particles were also able to activate primary murine B cells more effectively than RABV alone, supporting the conclusion that BAFF is functionally displayed on the surface of the virus particle. The incorporation of membrane-anchored BAFF into the virus particle improved the speed, magnitude and quality of vaccine-induced immunity as a live vaccine vector. Because BAFF is displayed on the surface of the virus particle and viral replication is not needed to produce BAFF, RABV-ED51-mBAFF showed potency as an inactivated RABV-based vaccine with as little as 125 ng/mouse of vaccine.

Current vaccines used to prevent rabies in humans rely on inactivated RABV strains. Pre-exposure vaccination is reserved for people at risk for infection, such as individuals working with rabies in diagnostic or research laboratories, veterinarians, and professional animal handlers. WHO also recommends that children in endemic areas receive pre-exposure vaccination because children under the age of 15 are disproportionately affected by RABV infections [3]. Despite these exceptions, the primary means of preventing rabies in humans relies on PEP administered after a person is exposed to a potentially rabid animal. WHO-recommended post-exposure treatment is complex and costly [45]. The US Centers for Disease Control and Prevention approved to reduce the number of inactivated RABV-based vaccine doses from five to four during human rabies post-exposure treatment in the U.S. [46]. WHO recommendations remain unchanged, although in areas of the world that are unable to afford this regimen, WHO recommends alternative vaccine schedules, most notably those used via intradermal inoculation or shortened regimens [47–49]. Despite the progress in developing alternative immunization schedules, they remain complex, expensive, multiple visits to medical facilities and skilled practitioners capable of administering the vaccine intradermally. These obstacles contribute to decreasing the widespread use and thus reducing the effectiveness of these vaccination regimens. Improving the efficacy of inactivated RABV-based vaccines might be key to prevent human deaths due to rabies.

Inactivated viral particles are generally poor immunogens because they do not elicit potent inflammatory responses required for effective CD4^+^ T-cell help. Inactivated RABV vaccines also generate a Th2-biased antibody response characterized by IgG1 antibodies in mice or IgG2 antibodies in humans [37] rather than more potent antiviral Th1-type antibodies. Modifications to current inactivated RABV-based vaccines might help to enhance their effectiveness in humans. The use of traditional adjuvants can improve antigen delivery and/or augment vaccine-induced immunity of various vaccines being tested in human clinical trials [50]. Indeed, studies describe preclinical data using CpG oligodeoxynucleotides (ODNs) [51, 52], poly(lactide-co-glycolide) microspheres [53], or GLA-SE [54] as adjuvants. These studies have suggested that the efficacy of inactivated RABV-based vaccines can be improved through the use of proper adjuvants. It is likely that as alternative adjuvants are developed and tested in the context of vaccines for other infectious agents, they can be developed for use with inactivated RABV-based vaccines.

In addition to traditional approaches to include vaccine adjuvants, incorporating molecular adjuvants into virus or virus-like particles is an option under investigation to improve vaccine-induced antibody responses. We previously showed that the incorporation of Intracellular Adhesion Molecule-1 (ICAM-1) into the membrane of RABV-based particles improved B cell activation ex-vivo and RABV-specific immunity *in-vivo* [20]. Others have shown that RABV-based VLPs displaying membrane-anchored GM-CSF [53], flagellin or Escherichia coli heat-labile enterotoxin B subunit [55] improve the efficacy of RABV-based vaccines. Membrane-anchored LTG, flagellin, cholera toxin B, or Ricin improve vaccine-induced immunity in the context of an inactivated influenza virus or influenza virus-based VLP vaccines [56–58]. Membrane-anchored molecular adjuvants also improve the efficacy of HIV/SIV-based vaccinations [59, 60]. Together, the incorporation of membrane-anchored molecular adjuvants has the potential to improve vaccine immunity against a wide range of infectious diseases. Of note, since replication is not needed to express the membrane-anchored molecular adjuvant in-vivo, these novel vectors can be utilized as inactivated vaccines. Indeed, we showed here that an inactivated RABV-based vector displaying BAFF improved the speed and magnitude of the anti-RABV antibody response without affecting the longevity of the response. This is critical since RABV-based vaccines are used in both pre- and post-exposure settings. Future studies will need to determine vaccine stability and evaluate mechanisms of attenuation. Nonetheless, these preliminary immunogenicity studies show that the incorporation of membrane-anchored molecular adjuvants into the surface of RABV particles holds promise to circumvent obstacles for the effective use of inactivated vaccines to prevent rabies infections in humans.
